# Impact of breast cancer on body image: a systematic review and meta-analysis

**DOI:** 10.1007/s00737-026-01754-7

**Published:** 2026-09-07

**Authors:** Serenay Yazici Sarikaya, Rui Wang, Ann-Christin S Kimmig, Sara Bruecker, Markus Hahn, Anna Wikman, Birgit Derntl

**Affiliations:** 1 Department of Psychiatry and Psychotherapy, Tübingen Center for Mental Health (TüCMH), University of Tübingen, Tübingen, Germany; 2 Department of Gynecology and Obstetrics, University of Tübingen, Tübingen, Germany; 3 Department of Women’s and Children’s Health, Uppsala University, Uppsala, Sweden; 4 German Center for Mental Health (DZPG), Partner Site Tübingen, Tübingen, Germany

**Keywords:** Breast cancer, Body image, Women’s health, Women’s mental health, Systematic review, Meta-analysis

## Abstract

**Background:**

Breast cancer is the most common cancer among females globally. While treatment is life-saving and essential, it can significantly affect physical health, psychological well-being, quality of life, and body image.

**Objectives:**

To systematically review and synthesize the evidence on the impact of breast cancer and its treatment on body image in females.

**Design:**

Systematic review and meta-analysis.

**Data sources and methods:**

Four major databases were searched for relevant peer-reviewed studies published until 2025. A total of 41 studies were included in the systematic review, of which 24 were eligible for the meta-analysis. Standardized mean differences with 95% confidence intervals were calculated using a restricted maximum likelihood model, with analyses conducted in JAMOVI and heterogeneity assessed via Q and I² statistics.

**Results:**

Females with breast cancer reported significantly lower body image scores than healthy controls (ES = -1.02). Furthermore, body image scores declined from pre-treatment to during treatment (ES = -0.45). Mastectomy was associated with poorer body image compared to breast-conserving therapy (ES = -1.11).

**Conclusion:**

Although medically essential, breast cancer treatment can adversely affect body image. Integrating body image support into treatment plans is crucial for promoting the overall health and quality of life of females diagnosed with breast cancer.

**Registration:**

PROSPERO No. CRD42024503033.

**Supplementary Information:**

The online version contains supplementary material available at https://doi.org/10.1007/s00737-026-01754-7.

## Background

Breast cancer (BC) was the most frequently diagnosed cancer type among females worldwide (Arnold et al. 2022, James et al. 2015, Jassim and Whitford 2013), with more than 2 million new cases reported in 2020 and over 300.000 additional cases recorded only in the USA in 2024 (Mallick and Sharma 2025, Ferlay et al. 2021). While BC treatment is life-saving and essential, research consistently demonstrates its impact across multiple aspects of well-being ( Brunet and Price, 2021), including mental and physical health, as well as body image (Brunet and Price 2021, Falk Dahl et al. 2010, Guedes et al. 2018). All of these factors can collectively influence and worsen the quality of life of afflicted females (Bakht et al. 2010, Fatehi et al. 2019, Luo et al. 2022, Tejeda et al. 2017, Begovic-Juhant et al. 2012, Piot-Ziegler et al. 2010).

BC diagnosis and its treatment can lead to a wide range of body-related side effects, each varying according to the type of treatment received. Similarly, the included meta-analyses comprised studies from Asia, the Middle East, the USA, and European countries, which may reflect sociocultural differences in appearance norms, femininity expectations, and social support, all of which can shape the experience and reporting of body image disturbances (Rasekh Jahromi et al. 2022, Cornell et al. 2017, Safarinejad et al. 2012). Radiation therapy (RT), while breast-conserving, introduces a distinct and multifaceted set of body image challenges that extend beyond the treatment period and it causing visible and direct physical changes to the breast and surrounding skin, including erythema, hyperpigmentation, desquamation, fibrosis and changes to breast shape and texture (Yoo et al. 2022, Lee et al. 2025, Chu et al. 2021). Beyond these physical changes, the repeated process of disrobing for treatment, which is required daily for several weeks, may itself heighten body awareness, self-consciousness and vulnerability, thereby contributing to body image disturbance (Boldrini et al. 2024, Mose et al. 2001, Boldrini et al. 2024). Chemotherapy is associated with systemic and highly visible changes, including hair loss, weight and appetite fluctuations, treatment-induced menopause and potential infertility (Cavalheiro et al. 2012). These changes can alter a woman’s sense of femininity and self-image. Endocrine therapy, which is often administered over an extended period, may also lead to musculoskeletal issues, sleep disturbances and sexual health concerns, such as reduced libido, vaginal dryness and pain (Sheffi et al. 2025, Can et al. 2008). These treatment-related changes can all affect how women with BC perceive their bodies, which can lead to an altered body image. Altered body image is a significant psychosocial concern for females with BC, with studies consistently reporting poorer body image compared to healthy controls (Rasekh Jahromi et al. 2022, Anagnostopoulos and Myrgianni 2009, Izydorczyk et al. 2019, Kraus 1999, Montañés-Muro et al. 2023, Savani et al. 2023). Ongoing treatment, in particular, has been linked to further declines in body image when comparing patients before and during treatment (Boquiren et al. 2015, Carver et al. 1998, Laroche et al. 2017, Metcalfe et al. 2015). Body image refers to the mental representation individuals form of their body, reflecting how they perceive their own physical appearance (Schilder 1935), and related concerns (Rasekh Jahromi et al. 2022). It has both personal and interpersonal dimensions, involving (dis)satisfaction with one’s physical appearance, (diminished) femininity, and (compromised) body integrity, as well as beliefs about how others perceive oneself (Fobair et al. 2003, Stokes and Frederick-Recascino 2003).

Given the pivotal role of treatment in shaping body image, it is essential to consider how different treatment modalities may differentially affect female’s perceptions of their bodies. Depending on tumor biology and cancer stage, treatment may include systemic therapy, radiotherapy, and surgery – either breast-conserving therapy (BCT) or mastectomy (MX) While BCT involves removal of the tumor and some surrounding tissue, MX entails complete removal of the breast. Studies comparing their effects on body image have yielded mixed results: some show more favorable outcomes with BCT (Arikan et al. 2020, Fung et al. 2001, Schou et al. 2005, Spatuzzi et al. 2016, Yilmazer et al. 1994), others report no significant differences (Alicikus et al. 2009, Yurek 1998), and one study even observed better body image in MX patients (Kraus 1999).

Recent literature has emphasized the need to advance our understanding of psychological adjustment in breast cancer by moving beyond symptom-specific or phase-limited perspectives toward integrative frameworks that capture mental health trajectories across the entire continuum of care. One such framework is the recently proposed Breast Cancer Psychological Integrative Phasic Model, which synthesizes existing theoretical and empirical work into a phase-sensitive structure encompassing screening, diagnosis, treatment, survivorship, and recurrence (Fortin et al. 2025). Each phase is marked by distinct psychosocial challenges and associated symptom profiles, ranging from anxiety and trauma-related distress to depression and fear of recurrence, highlighting how mental health evolves in parallel with medical treatment (Fortin et al. 2025, Wei et al. 2016). This phasic perspective provides a valuable lens for examining body image disturbances, which often arise or intensify during treatment and survivorship. Rather than treating body image problems in isolation, the model situates them within a broader constellation of psychological responses shaped by temporal and structural dynamics across the cancer trajectory (Fortin et al. 2025).

Overall, BC and its treatment modalities appear to lead to mixed results regarding body image, and a quantitative evaluation of the effects of disease, different treatment modalities, and treatment durations is still lacking. To our knowledge, this is the first systematic review and meta-analysis to simultaneously examine body image changes in these three domains: (1) disease effect, (2) treatment effect, and (3) treatment modality. Therefore, a systematic review and meta-analysis of the existing data on body image in females with BC is required to offer a comprehensive overview of the field. This will help guide future studies, directing scientific inquiry to explore any gaps in understanding and improve knowledge for physicians, patients, and their relatives.

The aim of the study is to investigate:

1. Effect of disease (females with BC vs. healthy controls): How does the body image of females with BC differ from that of healthy individuals?
2. Effect of treatment (before vs. during): What changes occur in the body image of females with BC when comparing the before to the ongoing treatment phase?
3. Treatment modality (MX vs. BCT): How do body image perceptions, including both negative and positive aspects, vary between females with BC undergoing mastectomy (MX) and those receiving breast-conserving therapy (BCT) (lumpectomy surgery)?

## Methods

A systematic review and meta-analysis on body image in females with BC was conducted and reported in accordance with the Preferred Reporting Items for Systematic Reviews and Meta-Analyses (PRISMA) 2020 statement (Page et al. 2021). Prior to performing this study, a review protocol was registered in the International Prospective Register of Systematic Reviews (PROSPERO) database (Registration No. CRD42024503033; https://www.crd.york.ac.uk/prospero/display_record.php? ID=CRD42024503033).

### Search strategy

Published studies up to 2025 were searched in PubMed, APA PsycINFO, CINAHL, and Web of Science Core Collection by Uppsala University Library. The following search terms were used: (“breast neoplasm*” OR “breast cancer*” OR “breast tumor*” OR “breast carcinoma*” OR “mammary cancer*” OR “mammary carcinoma*” OR “mammary tumor*” OR “mammary neoplasm*”) AND (“female*” OR “girl*” OR “woman*” OR “women*”) AND (“body image” OR “body acceptance” OR “body schema” OR “body dissatisfaction” OR “body esteem” OR “body image disturbance*” OR “body checking” OR “dysphoria” OR “physical self” OR “self-image” OR “body shame” OR “body perception” OR “body satisfaction” OR “body appreciation”). Please see the supplementary material for the complete search strategies.

### Eligibility criteria

Articles were considered eligible if they met the following inclusion criteria: (1) original research; (2) measuring body image as a primary outcome variable (a full list of questionnaires is provided in Supplementary Table 1); (3) fully accessible text; studies on female breast cancer patients vs. healthy controls, baseline vs. ongoing breast cancer treatment process, MX vs. BCT; (5) studies only cover current female breast cancer patients with no metastasis and no breast reconstruction; (6) observational studies. Articles were excluded when they met one of the following criteria: (1) non-peer-reviewed manuscripts, non-research articles, non-English articles, meeting abstracts; studies on physical, psychological, or psychosocial intervention/counseling; (3) studies on couple-based issues; (4) studies on unmet needs; (5) lack of a control group.

This study included only females with non-metastatic BC to increase validity and consistency, as metastatic BC occurs when cancer has spread beyond the breast, indicating an advanced stage, and is often associated with severe symptoms such as fatigue and pain, which can exacerbate treatment side effects (Butt et al. 2008, Palesh et al. 2007).

### Study selection and data extraction

Two reviewers (SYS and RW) independently screened the titles and abstracts of all retrieved studies based on the predefined eligibility criteria. Full-text articles were then assessed for eligibility. Any disagreements were resolved through discussion and consensus, and when necessary, a third reviewer was consulted. Subsequently, the two reviewers independently extracted relevant data from the included studies. Any discrepancies were identified and resolved through consensus in consultation with co-authors MH and BD. The following information was extracted for all articles: (1) Publication information (title/author/year/journal); (2) Study characteristics (aim/study design/data collection/mean and standard deviation); (3) Participants (sample size/age/treatment/symptom); (4) Outcomes (instrument-related data, mean results).

In the meta-analysis, mean and standard deviation values were directly extracted from the original tables in the studies. Transforming scale orientations (e.g., for positive vs. negative body image) was avoided to preserve data integrity, as such transformations could compromise the original meaning and reliability of the data reported by the primary sources. Various body image-related questionnaires were included in the meta-analysis (see Supplementary Table 1). Positive body image was defined by higher scores on relevant questionnaires, whereas negative body image was indicated by higher scores on specific questionnaires. As such, two separate meta-analyses were conducted for the comparison of MX vs. BCT groups.

If any articles included two groups/subgroups, data were extracted separately for each group/subgroup. When the study measured body image repeatedly (more than two times) in a longitudinal fashion, we included only two distinct measurement time points, each with a larger number of participants, to minimize the risk of result duplication and ensure the robustness of our analysis. When multiple studies included the same sample, only the study with the largest sample was included to avoid duplication of participants. Articles that described body image but lacked quantitative data – despite attempts to obtain this information from the authors via email – were included in the descriptive systematic review rather than the quantitative meta-analysis.

### Quality assessment

#### Assessment of risk of bias

The risk of bias for each included paper was evaluated independently by two reviewers using the Newcastle-Ottawa Scale (NOS) (Wells et al. 2014) and the Risk Of Bias in Non-randomized Studies of Interventions Scale (ROBINS-I) (Corbett et al. 2014) based on the study characteristics. 

The NOS is a straightforward quality assessment tool specifically for non-randomized cohort and case-control studies, focusing on three elements: selection of study groups, comparability between groups, and allocation of the exposure or outcome of interest. Assessment quality is categorized as low (0–4), moderate (5-7), or high (8-10). For some studies, it was not possible to apply the NOS, in these cases the ROBINS-I tool was applied. The ROBINS-I is a comprehensive tool suitable for various study designs, offering a nuanced quality assessment through detailed questions on potential biases, such as those in reported outcomes and intervention classification. Specifically designed for non-randomized intervention studies, ROBINS-I evaluates seven bias elements: confounding, participant selection, intervention classification, deviations from intended interventions, missing data, outcome measurement, and selection of reported outcomes. Quality is rated in four categories: low, moderate, serious, and critical.

#### Publication bias

The publication bias was assessed by visual inspection of funnel plots and Egger’s regression test for asymmetry (Egger et al. 1997) using the JAMOVI (2.3.38) software (Jamovi 2022). Significant publication biases were identified by p-values lower than 0.05.

### Statistical analysis

We analyzed the differences in body image between (1) females with BC vs. healthy controls, (2) females with BC before vs. during treatment, and (3) females with BC who had undergone MX vs. BCT. The standardized mean differences and their 95% confidence intervals (CI) were calculated using a Random Effects (RE) model with between-study variance estimated via Restricted Maximum Likelihood (REML) (PATTERSON HD 1971), due to substantial heterogeneity in clinical samples and methodological approaches between studies. Mean, standard deviation and sample size were provided from the included studies or corresponding authors. All statistical analyses were performed using JAMOVI (version 2.3.38) (Jamovi 2022), and the alpha level was set at *p* < 0.05. Forest plots present the overall effect size estimates along with their corresponding 95% confidence intervals. The number of included studies in each analysis was reported by “k” and effect size was reported by “µ”. Heterogeneity among the studies in each analysis was evaluated using Cochran’s Q and Higgins’ I^2^ statistics (Wells et al. 2014). A threshold of 25%, 50%, and 75% was used to differentiate between low, medium, and high heterogeneity.

## Results

### Literature retrieval results and study characteristics

**Fig. 1:**
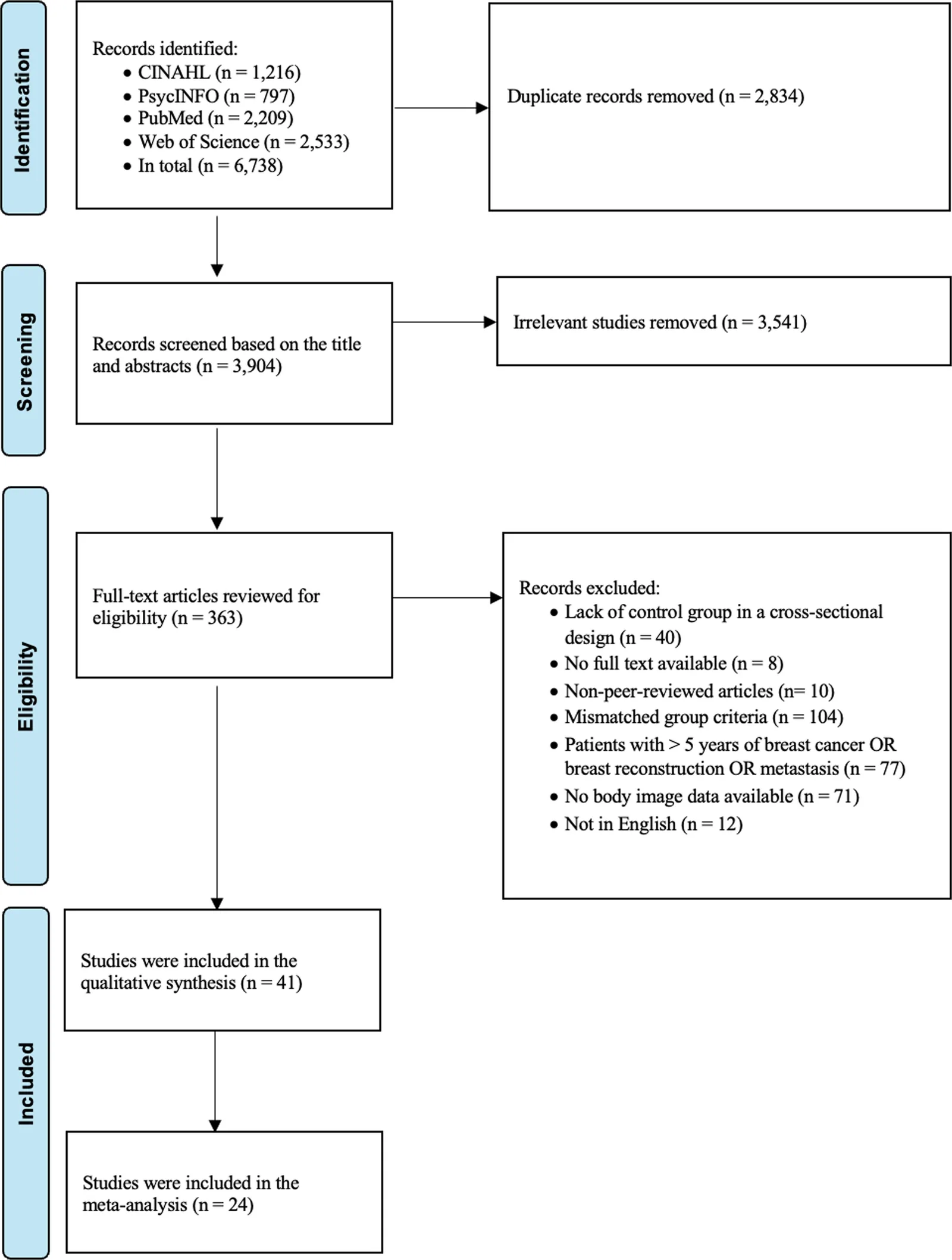
PRISMA flow diagram of the study search and selection process

The database search initially yielded a total of 6738 studies. After duplicate removal, 3904 records underwent title and abstract screening, of which 3541 were excluded and retained 363 studies for further evaluation. After the full-text screening, 322 studies were excluded. See Fig. 1 for a flow chart of article selection following the PRISMA 2020 guidelines.

Ultimately, 41 studies were included in the final analysis of this study. The included studies were published between 1988 and 2025. Based on the availability of body image scores, 24 body image-related studies contained adequate data for quantitative synthesis in the meta-analysis. Five reported differences between females with BC and controls, ten compared before vs. during treatment, six compared MX vs. BCT in the negative body image direction, and eight estimated MX vs. BCT in the positive body image direction. Some studies contributed data to multiple meta-analyses due to their design and reporting structure. Specifically, certain studies included both cross-sectional comparisons (e.g., females with breast cancer vs. healthy controls) and longitudinal assessments (e.g., pre-treatment vs. during treatment) or provided subgroup analyses based on surgical type (e.g., MX vs. BCT). For instance, a single study might compare females with BC to healthy controls while also reporting within-group changes over time among the breast cancer cohort, thereby contributing to a second meta-analysis. There were 4 studies that were included in more than one meta-analysis. Those studies marked in Tables 1, 2 and 3 indicate which data were used for which meta-analysis. These studies included 6872 females with BC and 398 healthy controls. The general characteristics of the 41 included studies are presented in Tables 1, 2 and 3 with the studies that could not be included in the meta-analysis highlighted with asterisks. 

**Table 1:** Characteristics of included studies investigating body image (BI) in female with breast cancer (BCP) and healthy controls (HC)

Study	CO	BCP										HC					Matched by age		BI Qu.		Quality of study
		*N*			Age(mean/ range)	BI score, mean (SD)	Tumor size/ Cancer stage		Treatment		Time of assessment	*N*	Age(mean/range		BI score,mean(SD)						
Aerts et al., 2014	BE	56			57/ 34–75	10.54 (8.17)	na/ Early BC stage		Surgery, Chemotherapy, Radiation therapy, Endocrine therapy		After treatment	81	56/ 26–80		4.49 (5.04)		Yes		BIS		NOS: 7
Kraus, 1999	USA	31			52/ 29–82	122.94 (3.33)	na/ Early BC stage		Surgery		na	30	53/ 29–82		116.47 (3.30)		na		BIS*		NOS: 4
Montañés Muro et al., 2023	SPA	27			54/ 38–70	0.78 (0.44)	na/ na		Surgery		After treatment	27	50/ 37–62		0.50 (0.27)		na		BIS		NOS: 7
Savani et al., 2023	IND	60			46/ na	10.48 (9.23)	na/ na		na		After treatment	60	46/ na		1.60 (2.49)		Yes		BIS		NOS: 7
Jahromi et al., 2022	IRI	111			48/ 30–55	51.56 (15.09)	Cancer grade 2		Chemotherapy, Radiotherapy, Surgery		After treatment	111	47/ 30–55		45.80 (16.82)		Yes		FOBIQ		NOS: 8
*Bagheri & Mazaheri, 2015	IRI	50			32/na	109.94(19.01)	na/na		Radiotherapy		After treatmen	50	33/X		120.20(19.82)		na		MBSRQ		NOS: 7
*Jablonski et al., 2018	PL	39			55/ 34–68	na	na		Surgery, BCT		After treatment	39	52/ 34–68		na		na		BS-Q		NOS:7
*Izdorczyk et al., 2019	PL	120			55/ na.	na	na		Mastectomy		After treatment	99	54/ n.a.		na		Yes		BAQ		NOS:4
*Anagnostopolous & Myrgianni, 2009	GR	70			na	na	na		Surgery, Chemotherapy, Endocrine therapy, Radiotherapy		After treatment	70	n.a.		na		na		BIS	NOS:7	

*SD* standard deviation,* BIS* body image scale (Hopwood et al. 2001), BIS* body image scale- modified version (Polivy et al. 1977), *MBSRQ* multidimensional body-self relations questionnaire (Cash 1990), *FOBIQ* fear of body image questionnaire (Littleton et al. 2005),* BS-Q *body self-questionnaire (Mirucka, 2017), *BAQ* body attitude questionnaire (Probst et al. 1995), *na*. missing data, *NOS* newcastle-ottawa scale. *BCT* breast conservative therapy, *BI QU* body image related questionnaires, * studies in only qualitative analysis, *CO* country, *BE* Belgium, *GR* Greece, *IN* India, *IR* Iran, *PL* Poland, *SP* Spain, *US* United States of America

**Table 2:** Characteristics of included studies investigating body image (BI) in baseline and during treatment females with breast cancer (BC)

Study	CO	Before treatment		During treatment		Age (mean/ range)	Tumor size/cancer stage	Treatment	BI QU	Qualityof study
		*N*	BI score, mean (SD)	*N*	BI score, mean (SD)					
Aerts et al., 2014	BE	81	1.53 (3.48)	66	4.05 (5.52)	57/ 34–75	na./ Early stage BC	Surgery, Chemotherapy, Radiotherapy, Endocrine therapy	EORTC QOL-BR23	NOS: 6
Bueno et al., 2018	BR	166	84.50 (23.40)	166	80.60 (26.80)	59/ na	na./ na	Surgery	EORTC QOL-BR23	NOS: 4
Browall et al., 2008	SE	75	90.00 (18.00)	65	66.00 (30.00)	64/ 55–80	na./ Stage I: 23 Other stages: 52	Chemotherapy, Radiotherapy, Endocrine therapy, Surgery	EORTC QOL-BR23	NOS: 6
Rahman et al., 2015	BD	250	86.00 (7.83)	250	70.46 (6.36)	44/ 21–67	T1: 15 T2: 102 T3: 53 T4: 80/ na.	Mastectomy, Neoadjuvant chemotherapy	EORTC QOL-BR23	NOS: 8
Viller et al., 2017	SPA	180	94.20 (13.20)	180	85.10 (23.00)	58/ na	na./ 0 : 22 I : 149 II : 128 III : 35 IV: 2	Chemotherapy, Radiotherapy, Hormone therapy, Biological therapy anti-Her2	EORTC QOL-BR23	NOS: 8
Binotto et al., 2020	BR	33	90.66 (14.99)	33	66.41 (31.49)	51/ na.	na./ Stage 1, 2 and 3	Surgery, Adjuvant or Neoadjuvant Taxane-based chemotherapy	EORTC QOL-BR23	NOS: 6
Castro e Silva et al., 2022	BR	80	25.73 (18.57)	80	34.06 (6.10)	52/ X	na./ Any at stage	Surgery, Chemotherapy	EORTC QOL-BR23	NOS: 5
Bahar et al., 2023	TUR	259	87.00 (18.00)	197	94.00 (13)	51/ 24–84	na./ I: 95 II: 156 III: 206	Radiotherapy, Surgery, Chemotherapy, Hormone therapy	EORTC QOL-BR23	NOS: 5
El Haidari et al., 2023	LB	120	89.20 (17.70)	116	81.00 (21.60)	54/ na.	T1: 42 T2: 71 missing: 7/ I: 52 II: 68	Surgery	EORTC QOL-BR23	NOS: 5
*Parker et al., 2007	USA	104	23.80 (5.50)	104	22.60 (6.10)	53/ na	na./ stage I and II	Chemotherapy, Radiotherapy, Surgery	EORTC QOL-BR23	NOS: 8
*Gadisa et al., 2021	ET	146	na	na	na	42/ na	I: 6 II: 48 III: 64 IV: 28	Chemotherapy	EORTC QOL-BR23	NOS: 7
*Laroche et al., 2017	FR	109	66.00 (33)	109	na	61/ na.	Early stage BC	Surgery, Chemotherapy, Aromatase inhibitor	EORTC QOL-BR23	NOS: 4
*Carver et al., 1998	USA	66	na	61	na	52/ 28–76	I: 49 II: 17	Surgery, Chemotherapy, Hormone therapy, Radiotherapy	MBA	NOS: 3
*Metcalfe et al., 2012	CA	24	na	24	na	53/ 30–83	0: 19 I: 26 II: 41 III: 14	Surgery, Radiotherapy	BIBC	NOS: 5

*SD* standard deviation, *BIS* body image scale (Hopwood et al. 2001), BIS* body image scale- modified version (Polivy et al. 1977), *EORT QoL-BR 23* european organization for research and treatment of cancer quality of life in patients with breast cancer (Sprangers et al. 1996), *BIBC* body image after breast cancer (Baxter, 1997), *MBA* measure of body apperception (Carver et al. 1998), *NOS* newcastle-ottawa scale, *na*. missing data, *BI QU* body image related questionnaires, * studies in only qualitative analysis, *CO* country, *CA* Canada, *FR* France, *ET* Ethiopia, *LB* Lebanon, *TUR* Turkey, *BR* Brazil, *SPA* Spain, *BE* Belgium, *BD* Bangladesh, *SE* Sweden

**Table 3:** Characteristics of included studies investigating body image (BI) in females with breast cancer (BCP) treated with breast-conserving therapy (BCT) and mastectomy (MX)

Study	CO	BCP with BCT				BCP with MX				Age(mean/ range)	BI QU	Qualityof study
		*N*	BI score mean(SD)	Tumor size/ cancer stage	Treatment	*N*	BI score mean(SD	Tumor size/ cancer stage	Treatment			
^a^Jendrian et al., 2017	GER	46	6.84 (6.30)	Tis/T1: 36 T2/T3/T4: 5	Surgery, Chemotherapy, Endocrine therapy	61	11.51 (7.67)	Tis/T1: 44 T2/T3/T4: 12	Surgery, Chemotherapy, Endocrine therapy	68/ 36.4–83.8	BIS	NOS: 6
^b^Jayasinghe et al., 2021	SL	19	58.35 (55.39)	T1: 15 T2: 27 T3 :5 T4 :2	Surgery, Adjuvant Chemotherapy, Hormone therapy, Adjuvant Radiotherapy, Axillary surgery	35	62.51 (27.68)	T1: 15 T2: 27 T3: 5 T4: 2	Surgery, Chemotherapy, Hormone therapy, Radiotherapy, Axillary surgery	58/ 36–81	EORT QOL-BR 23	ROBINS-I: MODERATE
^b^Munshi et al., 2010	IND	142	77.70 (21.80)	Early BC: 119 Advanced BC: 23	Adjuvant radiotherapy, Surgery	113	79.80 (35.20)	Early BC: 51 Advanced BC: 62	Adjuvant radiotherapy, Surgery	na	EORT QOL-BR 23	ROBINS-I: LOW
^b^King et al., 2000	AU	261	71.00 (15.00)	T1 and T2 (no exact number)/ Early stage of BC	Surgery, Radiotherapy, Chemotherapy	43	48 (28)	T1 and T2 (na)/ Early stage of BC	Surgery, Radiotherapy, Chemotherapy	BCT:54/25-81 MX:53	ESBC	NOS: 5
^b^Konieczny and Fal, 2023	PL	125	58.97 (32.22)	na.	Surgery, Radiotherapy, Chemotherapy, Hormonal therapy, Systemic therapy	64	58.73 (31.16)	na.	Surgery, Radiotherapy, Chemotherapy, Hormonal therapy, Systemic therapy	55/ na.	EORT QOL-BR 23	ROBINS-I: LOW
^b^Dubashi et al.,^2010^	IND	18	76.88 (33.03)	T1: 2 T2: 31 T3: 18	Surgery, Chemotherapy, Hormonal therapy, Bilateral salpingo-oopherectomy	33	82.38 (26.20)	T1: 2 T2: 31 T3: 18	Surgery, Chemotherapy, Hormonal therapy, Bilateral salpingo-oopherectomy	30/ na.	EORT QOL-BR 23	ROBINS-I: LOW
^b^Mock, 1988	USA	90	97.20 (12.31)	Early BC stage	Surgery, Adjuvant chemotherapy	62	98.20 (12.47)	Early BC stage	Surgery, Adjuvant chemotherapy	52/ 29–79	BIS	NOS: 4
^b^Jankowska-Polańska et al., 2020	PL	50	74.50 (24.99)	na./ na	Surgery, Radiotherapy, Chemotherapy, Hormonal therapy	50	57.39 (24.25)	na./ na	Surgery, Radiotherapy, Chemotherapy, Hormonal therapy	53/ na	EORT QOL-BR 23	ROBINS-I: LOW
^a^Ardakani et al., 2020	IRI	109	12.87 (6.41)	T1: 10 T2: 11 T3: 8 T4: 11 na: 140 na	Surgery, Radiotherapy, Chemotherapy, Hormonal therapy	71	15.18 (6.51)	T1: 10 T2: 11 T3: 8 T4: 11 na: 140 na	Surgery, Radiotherapy, Chemotherapy, Hormonal therapy	46/ 25–70	BIS	ROBINS-I: LOW
^a^Fonseca et al., 2014	PRT	26	3.69	na./ na	Surgery, Neoadjuvant chemotherapy	29	8.24 (7.22)	na./ na	Surgery, Neoadjuvant chemotherapy	54/ 32–79	BIS	ROBINS-I: MODERATE
^a^Aerts et al., 2014	BE	81	1.53 (3.48)	na./ na.	Surgery, Radiotherapy, Chemotherapy, Hormonal therapy	68	6.99 (7.58)	na./ na.	Surgery, Radiotherapy, Chemotherapy, Hormonal therapy	BCT: 57/ 34-75 MX: 54/ 26–74	BIS	NOS: 7
Montañés -Muro et al., 2023	SPA	13	0.64 (0.38)	na./ na	Surgery	14	0.91 (0.47)	na./ na.	Surgery	53/ 38–70	BIS	NOS: 7
^b^Parker et al., 2007	USA	104	23.6 (6.90)	na./ Stage I and II BC	Chemotherapy, Radiotherapy, Surgery	45	22.50 (5.10)	na./ Stage I and II BC	Chemotherapy, Radiotherapy, Surgery	BCT : 53 MX: 52/ na	MBSRQ	NOS: 8
^a^Shekhar et al.,^2023^	IND	402	2.78 (3.67)	T1: 215 T2: 187/ 0: 10 1: 72 2: 253 3: 34	Hormone therapy, Radiotherapy, Surgery	304	29.38 (11.55)	T2: 148 T3: 156/ 0: 24 3: 280	Hormone therapy, Radiotherapy, Surgery	< 35 : 28 35-55: 362 55-70: 266 > 70: 50	OBC	ROBINS-I: LOW
*Spatuzzi et al.,^2016^	ITA	72	na	BC stage 1–2	Surgery, Chemotherapy, Radiotherapy, Hormone therapy	44	na	BC stage 1 & 2	Surgery, Chemotherapy, Radiotherapy, Hormone therapy	BCT mean: 57 MX mean: 61.3	EORT QOL-BR 23	ROBINS-I: LOW
*Arican Alicikus et al., 2009	TUR	61	na	na	Surgery, Chemotherapy, Radiotherapy, Hormone therapy	51	na	na	Surgery, Chemotherapy, Radiotherapy, Hormone therapy	50/ 31–65	MBSRQ	ROBINS-I: LOW
*Yilmazer et al., 1994	TUR	40	na	BC stage 1& 2	Surgery, Chemotherapy, Radiotherapy	40	na	BC stage 1 and 2	Surgery, Chemotherapy, Radiotherapy	BCT: 40/ 27–50 MX: 43/ 30–50	A draw a person test	ROBINS-I: MODERATE
*Fung et al., 2001	CHN	17	na	Tis:1 T1:10 T2:6	Surgery	15	na	Tis:1 T1: 4 T2:!0	Surgery	BCT: 47 /na MX: 50 /na	Adopted initially the body image index	ROBINS-I: LOW
*Schou et al., 2005	NOR	85	na	Tis:2 T1:68 T2:30	Systematic therapy, Surgery, Radiotherapy	66	na	Tis:2 T1:68 T2:30	Systematic therapy, Surgery, Radiotherapy	56/ 21–78	CAWAC	ROBINS-I: LOW
*Yurek et al., 2000	USA	78	na	T2: 165 T3: 25	Surgery, Adjuvant therapy	79	na	T2: 165 T3: 25	Surgery, Adjuvant therapy	51/ 30–84	BSS	ROBINS-I: LOW
*Arikan et al., 2020	TUR	52	na	T1:9 T2: 71 T3: 19 T4: 18	Surgery, Chemotherapy, Target therapy, Hormone therapy	58	na	T1:9 T2: 71 T3: 19 T4: 18	Surgery, Chemotherapy, Target therapy, Hormone therapy	56/ 27–91	BIS	ROBINS-I: LOW
*Afshar-Bakshloo et al.,^2023^	GER	212	Na	T0: 161 T1: 48	Surgery, Chemotherapy, Target therapy, Radiotherapy	27	Na	T0: 27	Surgery, Chemotherapy, Target therapy, Radiotherapy		EORT QOL-BR 23	ROBINS-I: MODERATE
*Kraus, 1999	USA	16	na	Early BC	na	15	na	Early BC	Surgery	52/ 29–82	BIS*	NOS: 4

*BIS* body image scale (Hopwood et al. 2001), BIS*: body image scale- modified version (Polivy et al. 1977), *OBC* objectified body consciousness (McKinley and Hyde 1996), *MBSRQ* multidimensional body-self relations questionnaire (Cash 1990), *EORT QoL-BR 23* european organization for research and treatment of cancer quality of life in patients with breast cancer (Sprangers et al. 1996), *CAWAC* caring about women and cancer (Veronesi et al. 1999), *BSS* body satisfaction scale (Berscheid et al. 1973), *ESBC* enhanced body image scale for breast cancer (Kenny et al. 2000), A draw a person test (Machover 1949), Adopted initially the body image index (Lasry et al., 1987), *n.a.* missing data, *NOS* newcastle-ottawa scale, *ROBINS-I* risk of bias in non-randomised studies - of interventions, *BI QU* body image related questionnaires, ^*a*^ negative direction, ^*b*^ positive direction, * studies in only qualitative analysis, *CO* country, *GER* Germany, *NOR* Norway, *BE* Belgium, *IRI* Iran, *CHN* China, *ITA* Italy,* IND* India,* SPA* Spain,* PRT* Portugal, *PL* Poland, *SL* Sri Lanka, *TUR* Turkey, *AU* Australia

**Fig. 2:**
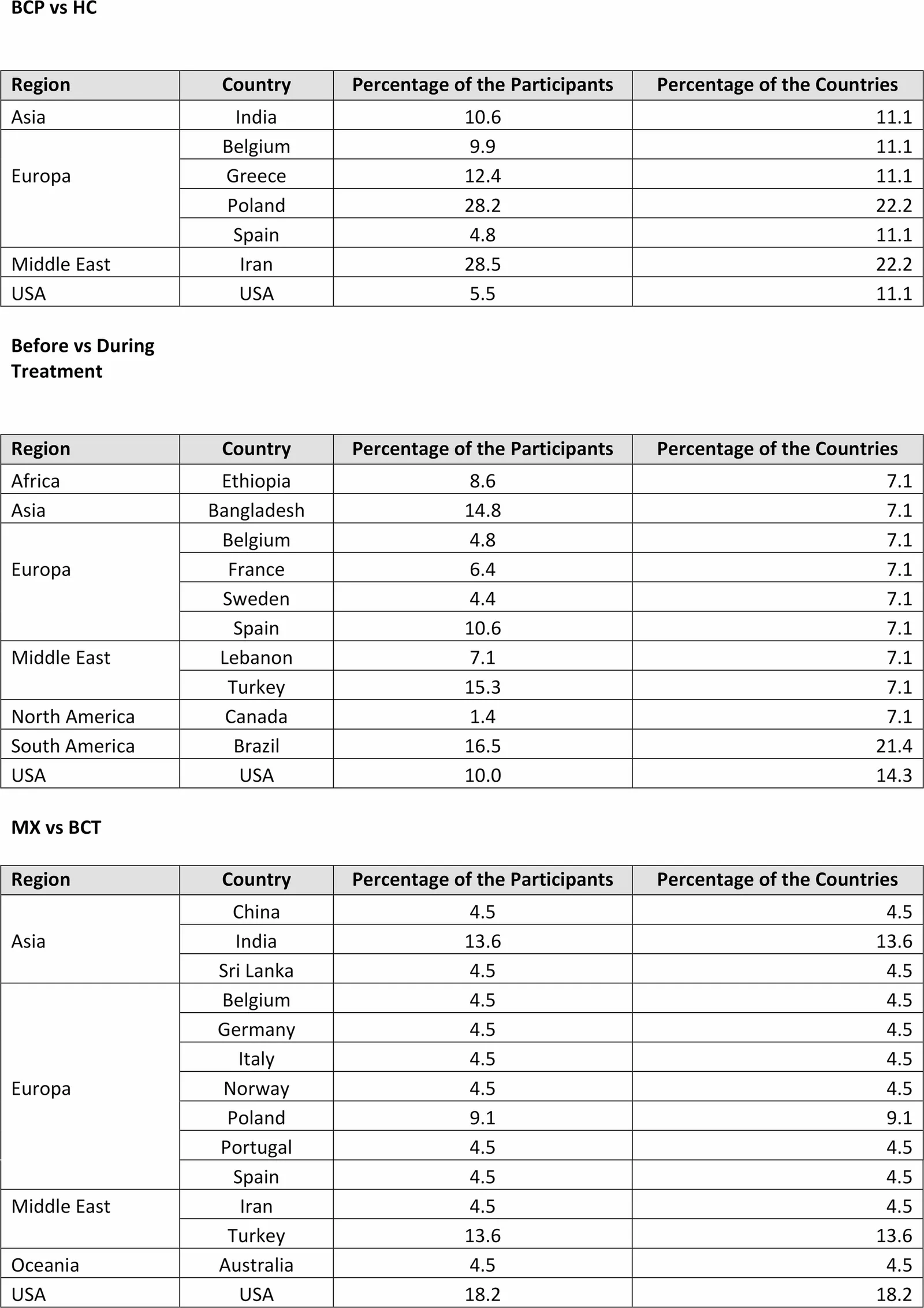
The table illustrates the percentage of countries and samples of each country included in the study

The geographic distribution of the studies included in the systematic review and meta-analysis is depicted in a table (Fig. 2). This figure highlights potential cultural influences on body image among females with BC. The full county breakdown per study is included in Tables 1, 2 and 3.

### Meta-analyses & qualitative analyses

#### Comparison of body image in females with BC vs. healthy controls

Nine articles were identified targeting this comparison and were published between 1999 and 2023. Among them, four studies lacked quantitative body image data and thus were included in the systematic review (see Table 1 for their characteristics).

Five studies were included in this meta-analysis and reported data from 469 females with BC and 479 healthy controls (with three studies using age-matched controls). They were conducted in Belgium, the USA, Spain, Iran, and India (Rasekh Jahromi et al. 2022, Kraus 1999, Montañés-Muro et al. 2023, Savani et al. 2023, Aerts et al. 2014).

**Fig. 3:**
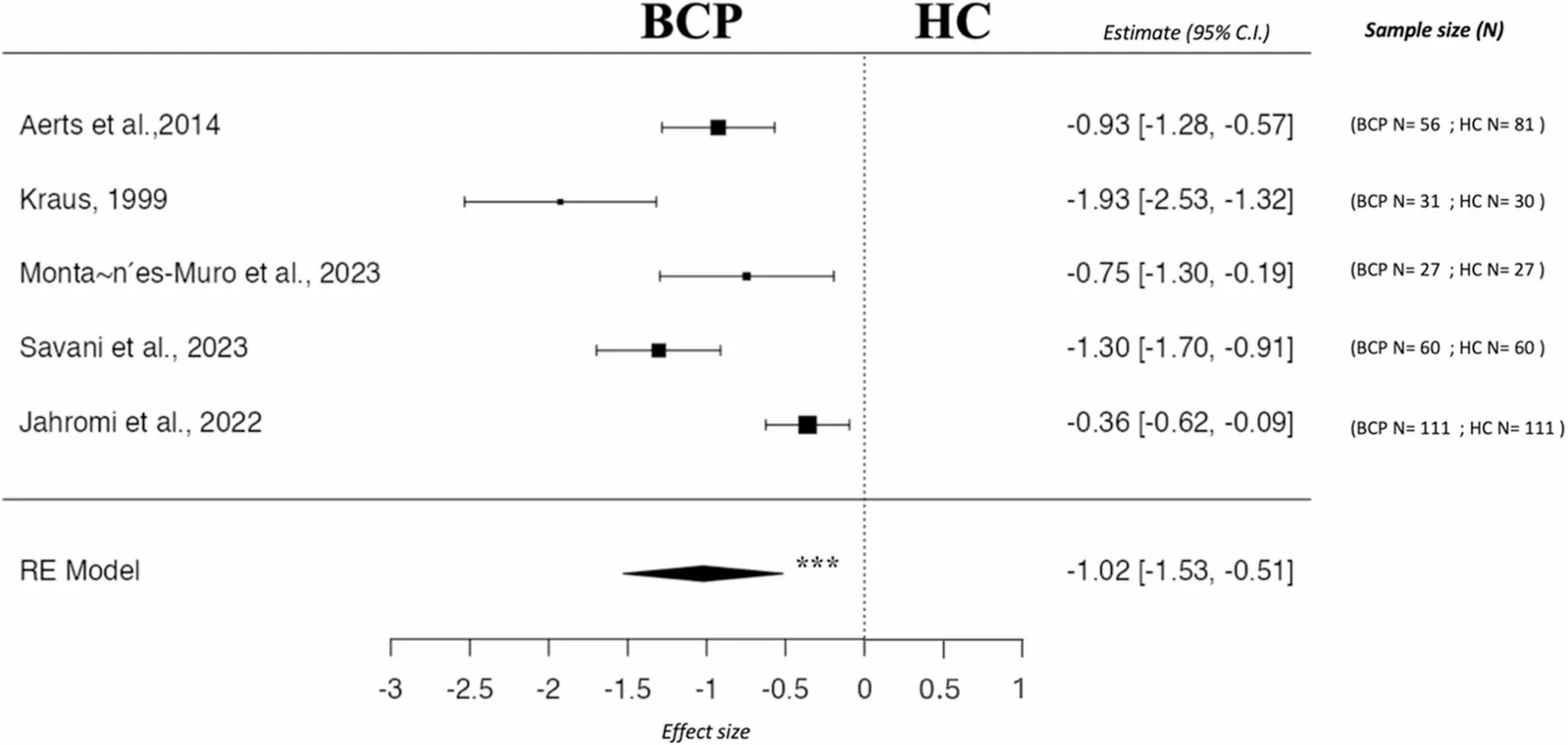
Forest plot of body image scores comparing values from females with breast cancer (BCP) and healthy controls (HC). Note. *** marks significant group difference with *p* < 0.001. Abbreviations: RE= Random Effects

Compared to healthy controls, females with BC had a significantly worse body image score (k = 5, µ = -1.02 95%CI [-1.53, -0.51], *p* < 0.001, see Fig. 3). This finding presented a large effect size in the negative direction (ES = -1.02). The heterogeneity between studies was high (I^2^ = 87.23%, *p* < 0.001).

##### Qualitative analysis (females with BC vs. healthy controls)

Studies report significantly higher levels of body dissatisfaction in females with BC than in healthy controls (Anagnostopoulos and Myrgianni 2009, Izydorczyk et al. 2019, Bagheri and Mazaheri 2015). Additionally, lower body acceptance was shown in females with BC compared to healthy controls (Jabłoński et al. 2018). These qualitative studies revealed that body image disturbances were most often associated with perceived changes in femininity and physical integrity, especially after a mastectomy. Women often reported dissatisfaction with their appearance, concerns about scars or breast asymmetry, and greater self-consciousness in social and intimate situations. These concerns were more pronounced in women who underwent a mastectomy.

#### Comparison of body image before vs. during treatment

Fourteen articles were identified targeting this comparison and were published between 1998 and 2023. Among them, four studies lacked quantitative body image data and thus were included only in the systematic review (see Table 2 for their characteristics).

Ten studies were included in this meta-analysis reporting data from 1262 females with BC before treatment and 1245 females with BC during treatment. The studies were conducted in Belgium, Turkey, Brazil (three studies), Sweden, Lebanon, Spain, the USA and Bangladesh (Aerts et al. 2014, Bahar 2023, Binotto et al. 2020, Browall et al. 2008, Bueno et al. 2018, Haidari et al. 2023, Marcelo Castro and Lúcia Penteado Lancellotti 2022, Parker et al. 2007, Rahman et al. 2015, Villar et al. 2017).

**Fig. 4:**
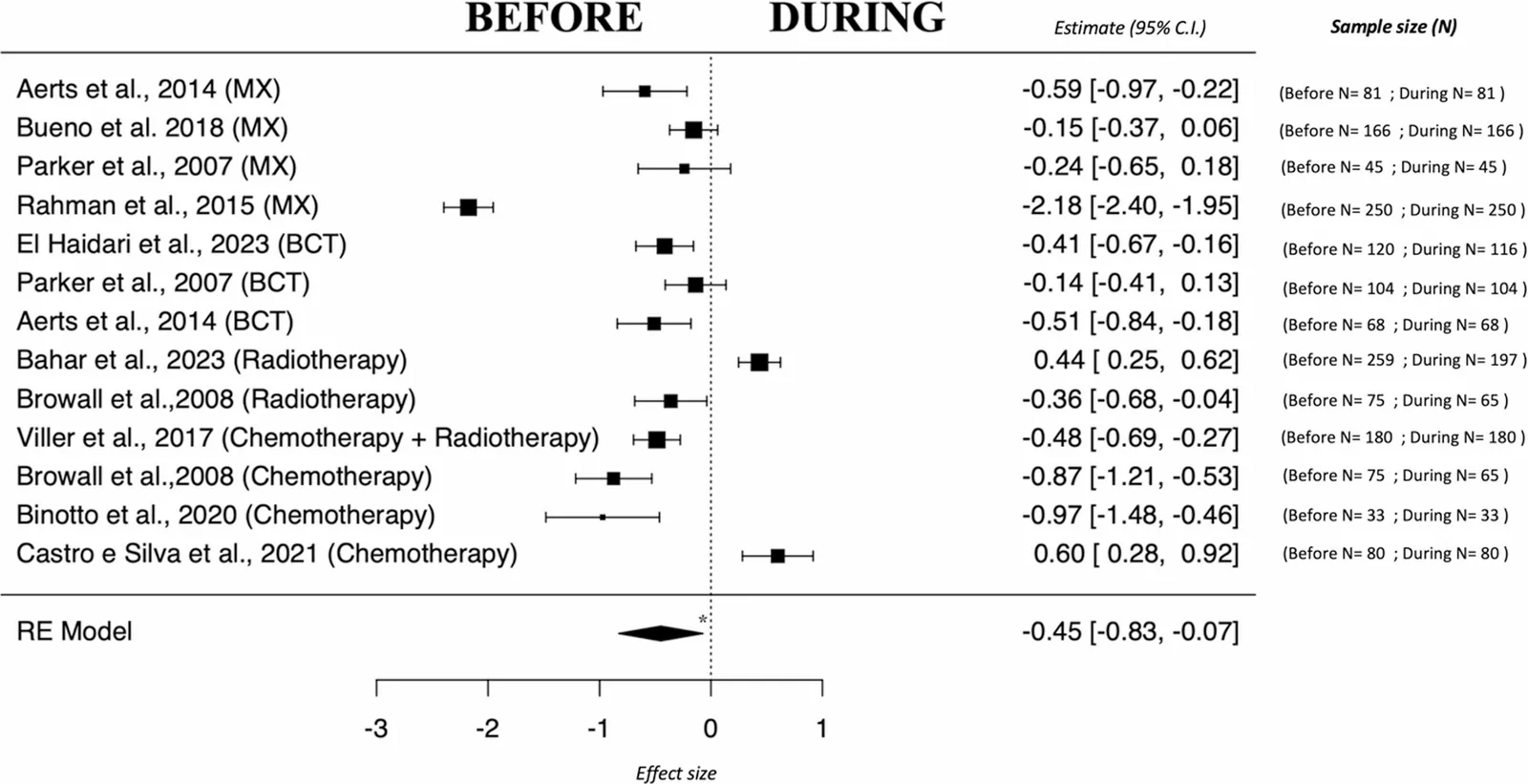
Forest plot of body image for the comparison between before vs. during treatment. Note. Treatment modalities are included in this forest plot. MX = mastectomy, BCT = breast-conserving therapy, * marks significant group difference with *p* < 0.05 Abbreviations: RE= Random Effects

Comparing body image data before vs. during treatment, the meta-analysis revealed that patients had significantly higher body image before the start of treatment than during treatment (k = 13 µ = 0.449, 95%CI [-0.83, -0.07], *p* = 0.019, see Fig. 4). This finding presented a small to moderate negative effect size (ES = -0.45). The heterogeneity between studies was high (I^2^ = 95.04%, *p* < 0.001).

##### Qualitative analysis (before vs. during treatment)

Three additional studies show that females with BC have a negative body image during treatment than before treatment (Carver et al. 1998, Laroche et al. 2017, Metcalfe et al. 2015). However, one study (Gadisa et al. 2021) reported no significant change in body image before vs. during breast cancer treatment. These qualitative studies revealed that BC treatment was consistently reported to affect body image. Commonly reported concerns included changes in perceived femininity, appearance-related distress, and difficulty adjusting to bodily changes, such as hair loss and breast size reduction compared to before treatment. Most studies indicated that these concerns were most evident during the early post-treatment period, and that aspects of body image disturbance, particularly those related to appearance and femininity, may persist even in long-term follow-up.

#### Comparison of MX vs. BCT- negative and positive direction

Twenty-two articles were identified targeting this comparison and were published between 1998 and 2023. Among them, eight studies lacked quantitative body image data and thus were included only in the systematic review (see Table 3 for their characteristics).

##### Meta-analysis of negative direction

Six studies were included in this meta-analysis that measured the negative direction of body image and relied on data from 677 BCT and 547 MX. They were conducted in Belgium, Iran, Portugal, Germany, Spain, and India (Montañés-Muro et al. 2023, Aerts et al. 2014, Fonseca et al. 2014, Jendrian et al. 2017, Shekhar et al. 2023, Yamani Ardakani et al. 2020)

**Fig. 5:**
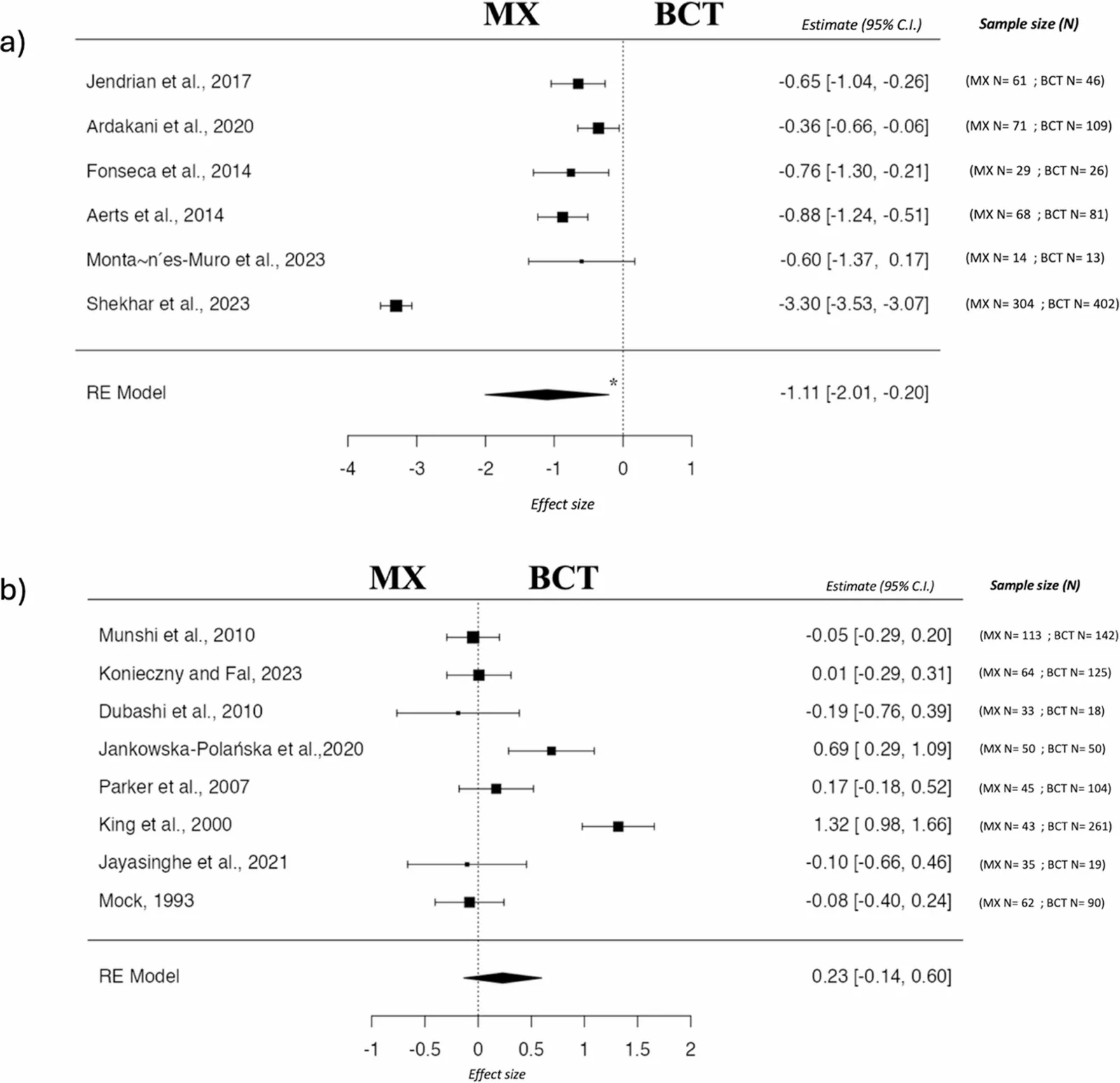
*a & b*: (**a**) Forest plot of negative body image for the comparison between mastectomy (MX) and breast conservative therapy (BCT) Note. Negative body image refers to a person’s dissatisfaction with their physical appearance or the way they perceive their body, * marks significant group difference with *p* < 0.05. (**b**) Forest plot of positive body image for the comparison between mastectomy (MX) and breast conservative therapy (BCT). Note. Positive body image means having a satisfactory and accepting attitude towards one’s body and appearance. Abbreviations: RE= Random Effects

Compared to the BCT group, the MX group had significantly higher negative body image (k = 6, µ = -1.105, 95%CI [-2.01, -0.20], *p* = 0.016, see Fig. 5a). This finding presented a large effect size in the negative direction (ES = -1.11). Again, the heterogeneity between studies was high (I^2^ = 97.19%, *p* < 0.001).

##### Meta-analysis of positive direction

Eight studies were included in this meta-analysis that measured the positive direction of body image and reported data from 809 BCT and 445 MX. Studies were conducted in India (two studies), Poland (two studies), Sri Lanka, Australia, and the USA (two studies) (Parker et al. 2007, Dubashi et al. 2010, Jankowska-Polanska et al. 2020, Jayasinghe et al. 2021, King et al. 2000, Konieczny and Fal 2023, Mock 1988, Munshi et al. 2010)

The meta-analysis revealed no significant difference between the BCT group and MX group, i.e., there was no group difference in terms of having a satisfactory and accepting attitude towards body image and appearance (k = 8, µ = 0.231, 95%CI [-0.14, 0.60], p = 0.216, see Fig. 5b). The heterogeneity between studies was high (I2 = 88.02%, p < 0.001).

##### Qualitative analysis MX vs. BCT

Six additional studies indicate that females with BC treated with BCT report higher body image than those in the MX group (Arikan et al. 2020, Fung et al. 2001, Schou et al. 2005, Spatuzzi et al. 2016, Yilmazer et al. 1994, Afshar-Bakshloo et al. 2023). However, Kraus et al. (Kraus 1999) found greater body image satisfaction in the MX group, while two other studies did not report a significant difference between the BCT and MX groups in either a positive (Alicikus et al. 2009) or negative (Yurek 1998) direction.

### Risk of bias

The risk of bias was assessed using NOS and ROBINS-I (see Tables 1, 2 and 3). Out of the twenty-five studies assessed by NOS, four were determined to be high quality (8-10), sixteen were of moderate quality (5-7) and five were judged as low quality (0–4) based on the NOS criteria. Out of the fifteen studies assessed by ROBINS-I, twelve were determined to be of high quality, and three were of moderate quality based on the ROBINS-I criteria.

### Publication bias

Publication bias was examined for all analyses of body image. The Egger’s tests were not significant for any of the analyses: females with BC vs. HC (k = 5, value = 1.781, *p* = 0.075), before vs. during treatment (k = 13, value = 0.373, *p* = 0.709), MX vs. BCT-negative (k = 6, value = 1.118, *p* = 0.263), and MX vs. BCT positive (k = 8, value = -0.471, *p* = 0.638 ) indicating no evidence for publication bias in these analyses.

## Discussion

In this study, we conducted a systematic review and meta-analysis to comprehensively examine the effects of BC and its treatments on body image. While breast cancer treatment is both life-saving and essential, it can have a significant impact on body image. Specifically, we addressed three key research questions: (1) How does the body image of females with breast cancer (BC) compare to that of healthy controls, (2) How does the body image of females with BC change before vs. during treatment, and (3) How does body image differ between patients undergoing mastectomy (MX) and breast-conserving therapy (BCT), considering both negative and positive aspects?

### Females with BC vs. healthy controls

The meta-analysis examined body image differences between females with BC and healthy controls, revealing significant body image dissatisfaction among females with BC. Similarly, qualitative analysis of studies included in the systematic review demonstrated the same pattern. Hence, the literature consistently shows that females with BC experience higher levels of body image dissatisfaction, body image-related problems, and lower body acceptance compared to females without BC (Bakht et al. 2010, Anagnostopoulos and Myrgianni 2009, Izydorczyk et al. 2019, Montañés-Muro et al. 2023, Savani et al. 2023, Fobair et al. 2003, Bagheri and Mazaheri 2015, Jabłoński et al. 2018). This negative body image may be attributed to treatment-related changes such as weight gain, hair loss, breast loss, and scarring, all of which can adversely affect body image (Montañés-Muro et al. 2023). Females with BC often perceive their bodies as a source of negative emotions, leading to lower self-esteem and difficulties in daily life (Jabłoński et al. 2018). These factors contribute to reduced body acceptance and more pronounced body image alterations. Both our findings and existing literature consistently show a strong link between poorer body image outcomes and the effects of cancer treatments, including chemotherapy, radiation therapy, endocrine therapy, and surgery. Meta-analysis is essential in uncovering deeper insights, as it not only consolidates evidence across studies but also helps address gaps and variability in the current research. This approach ensures a more thorough and reliable understanding of these treatment effects.

Currently, various types of treatments are chosen based on factors such as tumor size, cancer stage, and the patient’s condition. These treatments have many common side effects, but also unique ones. Chemotherapy-related hair loss, fertility concerns associated with endocrine treatments such as tamoxifen, and surgical procedures like mastectomy can all negatively affect how women perceive their bodies, femininity, and motherhood (Rasekh Jahromi et al. 2022, Cornell et al. 2017, Safarinejad et al. 2012, Cavalheiro et al. 2012, Sheffi et al. 2025, Can et al. 2008).

Radiation therapy (RT), however, deserves more specific attention in this context, as its effects on body image are often less emphasized. RT causes both short- and long-term changes that are directly visible in the treated breast and surrounding skin, with some of them persisting long after treatment, thereby serving as ongoing reminders of the illness experience (Yoo et al. 2022, Lee et al. 2025, Chu et al. 2021). Some of these changes may persist long after treatment and serve as ongoing reminders of the illness experience. In addition, RT requires women to attend regular treatment sessions, during these sessions, they need to repeatedly expose and undress the chest area in a clinical environment. This repeated exposure may increase feelings of vulnerability and discomfort and can contribute to body image concerns in ways that are somewhat different from other treatment modalities (Boldrini et al. 2024, Mose et al. 2001, Boldrini et al. 2024).

Importantly, the impact on body image frequently extends well beyond the active treatment phase. Research indicates that many females with BC continue to experience persistent, or even increased, body dissatisfaction during survivorship, as psychological adaptation often lags behind physical healing (Moreira and Canavarro 2010). Body image remains dynamic after treatment, influenced by enduring physical changes as well as emotional and social factors (Cernikova et al. 2024).

Psychosocial interventions have shown promise in supporting this adjustment, especially when aligned with individual characteristics and treatment experiences. A recent systematic review has shown that various interventions including cognitive behavioral therapy, mindfulness-based programs, self-compassion writing exercises, and body image–focused group therapies, can effectively reduce negative body image and promote positive aspects such as body appreciation and acceptance (Anne Thamar Louis et al. 2024). For instance, programs like “Accepting your Body after Cancer” and guided imagery sessions have been shown to alleviate distress and enhance psychological resilience in females with BC (Anne Thamar Louis et al. 2024, Lewis-Smith et al. 2018). Tailoring these interventions to individual characteristics, cancer stage, and cultural background can further increase their relevance and impact. Embedding such strategies into clinical pathways from diagnosis through survivorship may help bridge the gap between research and practice, ultimately improving the psychological well-being and quality of life of females with BC.

Body image has psychosocial dimensions that are shaped by social norms and culture (Bakhshi 2011). Research indicates that social norms significantly influence cancer-related perceptions in females with BC, often leading to negative body image (Thorne and Murray 2000). Anagnostopoulos and Myrgianni (Anagnostopoulos and Myrgianni 2009) report that in Western cultures, women’s bodies are highly valued, with breasts playing a crucial role in both gender identity and overall body integrity. Our systematic review and meta-analysis included studies not only from Western countries but also from regions such as the Middle East and Latin America (see Fig. 2). This highlights the global impact of breast cancer on body image, drawing on data from diverse cultural contexts rather than focusing solely on a Western perspective. Although subgroup analyses to directly compare cultural differences were not conducted, the inclusion of studies and participants from various countries provides valuable insights into potential variations across populations. This multicultural approach broadens the understanding of body image issues beyond the confines of Western culture, offering a more comprehensive and inclusive perspective in the field.

Emerging evidence suggests that culture not only contextualizes but also actively shapes how females experience body image following breast cancer. Yoo et al. highlight the role of sociocultural factors (such as education, economic background, and societal expectations) as key influences on body image distress in individuals with cancer (Yoo et al. 2024). A recent systematic review further underscores that appearance-related norms, gender roles, and cultural values related to femininity can shape coping mechanisms and perceptions of bodily change (Abdoli et al. 2024). For instance, in collectivist societies, physical integrity may be closely tied to familial or marital roles, potentially intensifying body image concerns, whereas in individualistic societies, self-presentation and autonomy may be more strongly emphasized. These dynamics suggest that cultural context can act as a moderator in the relationship between cancer treatment and body image outcomes. Future meta-analyses should examine these differences systematically, using culturally sensitive measures and conceptual frameworks.

Puberty, pregnancy, and menopause are crucial hormonal transition phases in a female’s life, each characterized by distinct physiological and psychosocial changes that can significantly influence body image (Erbil 2018). These transitions may be particularly complex in the context of BC, as the disease and its treatments can intersect with and disrupt these developmental stages (Anastasiadi et al. 2017, Johnson 2001, Mathur and Nath 2022), adding complexity to simple comparisons of patients with healthy controls. For example, adolescents and young women with cancer may face unique challenges related to identity formation, peer comparison, and body image during puberty and emerging adulthood (Vani et al. 2021, Paterson et al. 2015, Saris et al. 2022). Fertility concerns and potential treatment-related infertility can further exacerbate distress and negatively affect body image in reproductive-age women during pregnancy-related transitions (Wytiaz et al. 2024, Sheffi et al. 2025). Conversely, treatment-induced or premature menopause in younger patients can intensify menopausal symptoms and accelerate age-related bodily changes, leading to further body image concerns (Sheffi et al. 2025, Rosenberg and Partridge 2013, Howard-Anderson et al. 2012). Therefore, examining body image during these transitions and exploring the experiences of females with BC in these phases, is crucial to account for possible variability in body image perceptions among different groups of females with BC.

### Treatment effects

The meta-analysis evaluated potential changes in body image before vs. during treatment, mainly including data from studies on chemotherapy (*n* = 4), radiotherapy (*n* = 3), and surgery (*n* = 7). The analysis confirmed that before the start of treatment, females with BC had a significantly higher body image scores, i.e. more positive body image, than during treatment (Kraus 1999, Carver et al. 1998, Laroche et al. 2017, Metcalfe et al. 2015, Aerts et al. 2014, Binotto et al. 2020, Rahman et al. 2015, Villar et al. 2017, Montazeri 2008). In terms of different treatment modalities, studies have shown a significant decrease in body image in females with BC undergoing chemotherapy (Perrone et al. 2015), radiotherapy (Browall et al. 2008), and post-surgery (Bueno et al. 2018, Rahman et al. 2015). Thus, independent of treatment modality, a decrease in body image was reported. These changes may be attributed to factors such as hair and breast loss, weight fluctuations, and scarring associated with the various treatment approaches (Montañés-Muro et al. 2023).

Interestingly, (Haidari et al. 2023) observed that married females with BC in Lebanon experienced a significant improvement in body image one day after breast-conserving surgery. Authors speculate that marital status may positively influence body image perceptions after surgery, possibly due to increased emotional resilience or supportive relationship dynamics within Lebanese culture (Haidari et al. 2023).

A qualitative analysis of the studies included in the systematic review revealed varying findings: while some studies (Carver et al. 1998, Laroche et al. 2017, Metcalfe et al. 2015) supported the meta-analysis results, others found no significant differences in body image between the before and during treatment periods (Parker et al. 2007, Gadisa et al. 2021). This inconsistency may be explained by a shift in focus from physical appearance to survival concerns during treatment. Depending on patient characteristics, they may prioritize different factors affected by BC, such as physical appearance, loss of fertility, unhealthiness, or high responsibilities in their lives (Parker et al. 2007). Another possible reason is that in these females with BC, body image is not affected by the side effects of treatment, but rather by the impact of being diagnosed with BC (Yurek 1998, Jayasinghe et al. 2021). For this reason, changes in body image need to be assessed carefully and external factors should be taken into account.

### Treatment modality (negative & positive direction)

MX and BCT are two primary treatment options for breast cancer (BC) (Fisher et al. 1989). This meta-analysis investigated the differences in body image between females with BC who underwent MX and those who had BCT. It was hypothesized that the various effects of these two different treatment modalities on the breast, such as scarring or complete breast removal, affect body image differently. It was expected that MX would have a more negative effect on body image compared to BCT. The results indicate that MX is associated with significantly greater negative body image than BCT. However, in terms of the positive aspects of body image, no significant group differences were found. Recent literature presents inconsistent findings regarding body image following these two treatment options: while several studies indicate that females with BC who underwent MX report worse body image than those undergoing BCT (Anagnostopoulos and Myrgianni 2009, Fung et al. 2001, Schou et al. 2005, Spatuzzi et al. 2016, Yilmazer et al. 1994, Fonseca et al. 2014, Jendrian et al. 2017, Yamani Ardakani et al. 2020, Konieczny and Fal 2023, Munshi et al. 2010, Afshar-Bakshloo et al. 2023, Jankowska 2014), others showed no significant difference (Alicikus et al. 2009, Yurek 1998, Dubashi et al. 2010, Jayasinghe et al. 2021). Previous studies observed greater body stigma (Baxter et al. 2006) and greater alteration to their body image (Spatuzzi et al. 2016, Härtl et al. 2003) in the MX group. Studies have shown that the MX group faces challenges with clothing and overall appearance, as well as body shame due to the loss of their breast(s) (Bartelink et al. 1985, Engel et al. 2004), which may contribute to greater body concerns and alteration.

Conversely, Kraus found that females with BC who underwent MX reported higher body image satisfaction compared to those who underwent BCT. The author suggested that, in life-threatening situations following a BC diagnosis, physical appearance may become less significant, and the MX group might experience a sense of relief from the cancer diagnosis, which could mitigate concerns about body image despite the loss of their breast(s) (Kraus 1999). While this interpretation provides valuable insights, it must be viewed with caution due to the small sample size in the study (MX = 15, BCT = 16) conducted in 1999. Furthermore, since the study was conducted over two decades ago, social perspectives, cultural attitudes, and advancements in treatment modalities may have significantly evolved, potentially influencing body image experiences differently in contemporary populations. This is particularly relevant considering that BCT techniques were not widely adopted until the mid-1990s, and the quality of these procedures has improved over time. It is also important to note that the studies evaluating BCT are not randomized, which could affect the reliability of the findings.

Furthermore, several studies suggest no significant differences in body image between patients undergoing MX or BCT (Alicikus et al. 2009, Yurek 1998, Dubashi et al. 2010, Jayasinghe et al. 2021). This may be due to individual differences and personal beliefs of females with BC, suggesting that body image concern may be influenced more by the cancer itself than by the type of treatment received (Alicikus et al. 2009, Yurek 1998, Dubashi et al. 2010, Jayasinghe et al. 2021). Furthermore, the lack of significant differences in body image outcomes between these two types of surgery underscores the importance of prioritizing body image concerns for all patients, regardless of the surgical approach (Jabłoński et al. 2018, Fobair et al. 2006).

Moreover, the mixed findings regarding the effects of BC and its treatment on body image highlight the need for further research that considers sociodemographic factors. Education level, marital status, age, treatment modalities, and patients’ own perspective on their illness may play an important role in their body image and perspective on cancer. Additionally, raising awareness about breast cancer, its treatment, and its potential impact on body image is essential to support patients in managing these challenges effectively. Providing psychoeducation to help patients manage potential side effects can improve their understanding of body image issues associated with changes in breast shape or loss after surgery and their ability to cope with BC (Tunç et al. 2023, Younis et al. 2021).

This systematic review and meta-analyses are the first to offer a comprehensive multi-parameter quantitative synthesis of the impact of BC on body image. Our rigorous inclusion criteria and standardized review process, in accordance with following the PRISMA guidelines, contribute to the robustness and reliability of our findings.

### Study limitations

Several limitations should be considered when interpreting these results. First, the number of studies included in some analyses was relatively small, particularly in the comparison of body image between breast cancer patients and healthy controls (*n* = 5). Second, the lack of differentiation between participants based on treatment duration (e.g., days, months, or years) is another limitation. Future studies should consider categorizing participants into multiple groups based on treatment duration to allow for a more nuanced understanding of the effects of treatment. Third, while most studies comparing females with BC to healthy controls collected data post-treatment, two studies included both pre- and post-treatment patients without distinguishing between these subgroups in their analyses or conclusions. This methodological limitation may have obscured potential differences in body image experiences across treatment stages. Additionally, the wide time span of the included studies (approximately 24 years) is an important limitation that should be considered. Substantial advances in breast cancer management occurred during this period, including the widespread adoption of breast-conserving surgery, improvements in surgical techniques, and refinements in radiotherapy delivery and chemotherapy regimens. These developments in treatment may have introduced systematic variability in body image outcomes across studies and should be considered when interpreting the findings. Breast-conserving surgery became widespread in the mid-1990s, with high-quality procedures becoming standard only after 2000. Poor surgical quality before 2000 likely influenced body image outcomes, but because only one study before 2000 included both MX and BCT, sub analysis was not possible. Lastly, since surgical studies are not randomized, postoperative satisfaction may not be differentiated without any bias.

The considerable heterogeneity observed in all our meta-analyses can be attributed to several interrelated factors, including differences in patient characteristics, treatment protocols, and study methodologies across the included studies. Variability in age, disease stage, cultural background, and psychosocial factors likely influenced how body image was perceived and reported by participants. In addition, the high heterogeneity observed across comparisons may have been driven by differences in treatment modality, timing of assessment, and cultural context. For example, the “during treatment” category likely combined studies assessing patients at very different clinical time points, ranging from immediately after surgery (*n* = 4) to active chemotherapy (*n* = 4) or ongoing radiotherapy (*n* = 2), which may have contributed to substantial variability in body image outcomes. Similarly, the included meta-analyses comprised studies from Asia (*n* = 1), the Middle East (*n* = 2), the USA (*n* = 1), Africa (*n* = 1), and European countries (*n* = 4), which may reflect sociocultural differences in appearance norms, femininity expectations, and social support, all of which can shape the experience and reporting of body image disturbances. Moreover, for the BCP vs. HC and before vs. ongoing comparisons, several studies included patients receiving different treatments, such as chemotherapy, radiotherapy, surgery, and endocrine therapy, within the same analysis, and subgroup analyses were not feasible to determine if these treatment modalities had different effects on body image. Methodological factors, including the use of diverse body image assessment tools (e.g., different questionnaires), study designs (e.g., cross-sectional or longitudinal), and small sample sizes, may also play a significant role in driving heterogeneity. Different body image instruments may capture distinct dimensions of the construct, such as appearance satisfaction, body concerns, and femininity, which may have further contributed to the variability in findings across studies For example, the Body Image Scale (BIS, *n* = 9) and the scale of the European Organization for Research and Treatment of Cancer (EORTC, *n* = 15) were the most frequently used instruments. Moreover, the EORTC was applied in all studies in the meta-analysis regarding the comparison of before vs. ongoing treatment. However, subgroup analyses with *n* > 3 were not possible because studies were grouped in different conditions (BCP vs. HC and MX vs. BCT). These variations might likely underlie the observed differences in body image outcomes among BC patients, resulting in significant heterogeneity across studies.

### Clinical implications and future direction

This systematic review and meta-analysis offer valuable insight into body image changes associated with BC and its treatment, providing a detailed scientific perspective on these effects.

Recognizing the complex effects of breast cancer and its treatment helps both patients and healthcare providers anticipate body image-related difficulties that may arise during the treatment process. This awareness can support the integration of body image considerations into routine care, patient communication, and education. The findings, therefore, underline the importance of addressing body image within psychoeducation and treatment planning to improve overall support and quality of life.

In clinical practice, identifying and managing body image-related concerns often depends on the structure and functioning of the multidisciplinary care team. While oncologists focus primarily on disease management, nurses, psycho-oncology professionals, and other allied health staff may play a more active role in recognizing and responding to emotional distress and concerns about body image (Ranaut et al. 2024, Nikita et al. 2022). Clinicians who have frequent and ongoing contact with patients during treatment, such as radiation therapists, are particularly well-positioned to observe early changes and initiate timely conversations when concerns arise. Embedding simple screening approaches and clearly defined referral pathways into routine oncology care could help ensure that body image concerns are identified and addressed more consistently, rather than relying on patients to initiate these discussions. Thus, establishing such structured pathways may reduce the risk of overlooking these important psychosocial issues in clinical practice.

Future research should reduce heterogeneity by standardizing assessment tools, stratifying participants by key variables, and harmonizing protocols to enhance comparability and deepen understanding of the relationship between breast cancer, its treatments, and body image.

## Conclusion

This meta-analysis offers valuable insight into body image changes associated with BC and its treatment, providing a detailed scientific perspective on these effects. The findings demonstrate that females with BC experience significantly poorer body image compared to females without BC. Additionally, BC patients report better body image before starting treatment than ongoing treatment phases, suggesting that treatment itself contributes to body image concerns. Furthermore, among females with BC, those who undergo MX tend to report worse body image outcomes compared to those receiving BCT, highlighting the differential impact of treatment types on body perception. Although BC treatment is essential, its impact on body image remains a significant challenge. This review highlights the need for comprehensive treatment that addresses these concerns to improve women’s overall well-being and quality of life.

## Supplementary Information

Below is the link to the electronic supplementary material.

Supplementary Material 1

## Data Availability

The data supporting the findings of this study are available from the corresponding author upon reasonable request.

## Abbreviations

BC: Breast Cancer
BCT: Breast Conserving Therapy
CI: Confidence intervals
HC: Healthy Controls
MX: Mastectomy
NOS: Newcastle-Ottawa scale
PRISMA: Preferred Reporting Items for Systematic Reviews and Meta-Analyses
PROSPERO: International Prospective Register of Systematic Reviews
RE: Random Effects
REML: Restricted Maximum Likelihood
ROBINS-I: Risk Of Bias in Non-randomized Studies of Interventions Scale
